# Dietary Supplements in People with Metastatic Cancer Who Are Experiencing Malnutrition, Cachexia, Sarcopenia, and Frailty: A Scoping Review

**DOI:** 10.3390/nu14132642

**Published:** 2022-06-26

**Authors:** Jolyn Johal, Chad Yixian Han, Ria Joseph, Zachary Munn, Oluwaseyifunmi Andi Agbejule, Fiona Crawford-Williams, Matthew P. Wallen, Raymond J. Chan, Nicolas H. Hart

**Affiliations:** 1 Caring Futures Institute, College of Nursing and Health Sciences, Flinders University, Bedford Park, SA 5042, Australia; jolyn.johal@flinders.edu.au (J.J.); chad.han@flinders.edu.au (C.Y.H.); ria.joseph@flinders.edu.au (R.J.); andi.agbejule@flinders.edu.au (O.A.A.); fiona.crawfordwilliams@flinders.edu.au (F.C.-W.); matthew.wallen@flinders.edu.au (M.P.W.); raymond.chan@flinders.edu.au (R.J.C.); 2 Joanna Briggs Institute (JBI), The University of Adelaide, Adelaide, SA 5001, Australia; zachary.munn@adelaide.edu.au; 3 Cancer and Palliative Care Outcomes Centre, School of Nursing, Queensland University of Technology, Kelvin Grove, QLD 4059, Australia; 4 School of Science, Psychology and Sport, Federation University, Mount Helen, VIC 3350, Australia; 5 Exercise Medicine Research Institute, School of Medical and Health Sciences, Edith Cowan University, Joondalup, WA 6027, Australia; 6 Institute for Health Research, The University of Notre Dame Australia, Fremantle, WA 6160, Australia; 7 Precision Medicine (Cancer), South Australian Health and Medical Research Institute, Adelaide, SA 5001, Australia

**Keywords:** dietary supplements, metastatic cancers, malnutrition, cachexia, sarcopenia, frailty, weight loss

## Abstract

Cancer-associated malnutrition, or cachexia, stemming from cancer or its treatments, is particularly prevalent in metastatic cancers, and is often interrelated with sarcopenia and frailty. Evidence suggests that dietary supplements play a role in managing these conditions. As metastatic cancer cells are associated with notable genomic and phenotypic alterations, response to dietary supplements may differ between metastatic and non-metastatic cancers. However, research in this area is lacking. This scoping review aims to identify the dietary supplements that have been studied in patients with metastatic cancers and malnutrition-related conditions, along with their proposed effects, mechanisms, outcome measures, and tools used. A systematic search was conducted across databases, including MEDLINE, EMBASE, CINAHL, and clinical trial registries. Of the initial 6535 records screened, a total of 48 studies were included, covering a range of dietary supplements—vitamins, minerals, antioxidants, proteins, amino acids, fatty acids, fiber, and others. While the types of dietary supplements included varied across cancer types, omega-3 and carnitine were investigated most often. Proposed relevant attributes of dietary supplements included their antioxidant, anti-inflammatory, anti-cancer, and immunomodulatory properties. Overall, there was a paucity of interventional studies, and more randomized controlled trials are warranted.

## 1. Introduction 

Cancer treatments have significantly improved over time, leading to a prolonged survival time after a diagnosis of metastatic cancer, and consequently an increased number of advanced cancer survivors [1]. Advanced cancer survivors experience a range of complications arising from cancer, its treatments, and metabolic derangements [2,3,4,5,6,7]. Diet-related unmet care needs, including a lack of appetite and gastrointestinal symptoms, are commonly reported among people with metastatic cancers [8].

Malnutrition, cachexia, and sarcopenia are highly prevalent in people with metastatic cancer. Approximately half of people with metastatic cancer are reported to be moderately to severely malnourished [9,10], and almost all require some form of nutritional intervention [9]. Cancer-associated malnutrition, or cachexia, is a weight loss syndrome involving systemic inflammation and complex metabolic processes, often due to reduced nutritional intake owing to cancer-related side effects [11,12]. Sarcopenia is defined by the presence of low muscle strength, low muscle quantity and quality, and poor physical performance [13]. Sarcopenia is frequently observed in people with metastatic cancer and is associated with poorer prognosis and outcomes, as compared to those without [14,15,16]. Meanwhile, frailty is increasingly recognized as a critical health issue in people with metastatic cancer [17]. This state of vulnerability can be a culmination of aging, life prolonging cancer treatments, and cancer itself, and is an independent predictor of mortality [18,19]. Malnutrition, cachexia, sarcopenia, and frailty exhibit phenotypically similar features that are interrelated [20].

Modifications to diet, such as the use of dietary supplements, can alleviate cancer-associated symptoms [21] and, at times, enhance the efficacy of cancer treatments [22]. Dietary supplements are defined as concentrated sources of nutrients or other ingredients with a nutritional or physiological effect [23]. These include vitamins, minerals, botanicals or herb extracts, amino acids, essential fatty acids, and fiber [23,24]. Although there are a lack of clinical guidelines or consistent recommendations for the use of dietary supplements in people with metastatic cancer, dietary supplements are reportedly used by cancer survivors with the intention of improving symptoms and outcomes [25,26]. A previous systematic review investigating the role of vitamins, minerals, proteins, and other supplements for the treatment of cancer cachexia reported insufficient evidence advocating for the use of dietary supplements in people with cancer [27]. However, the review did not include younger people or people with malnutrition-related conditions other than cachexia (e.g., sarcopenia and frailty), and inclusion criteria were limited to cancer survivors with cachexia who may not necessarily have metastatic cancer. As metastatic cancer cells are associated with significant genomic and phenotypic alterations, responses of people with metastatic cancer to dietary supplements may differ from people with non-metastatic cancer and earlier stages of disease [2]. There is currently a lack of evidence synthesis that has systematically explored different dietary supplements that have been used in studies conducted in people with metastatic cancer and malnutrition-related syndromes (e.g., cachexia, sarcopenia, and frailty).

To address this gap, the primary aim of this scoping review was to systematically map out the body of evidence regarding dietary supplements administered orally or enterally in patients with metastatic cancers and malnutrition-related syndromes, including their hypothesized effects, proposed mechanisms, as well as outcome measures (and corresponding tools) used to evaluate their effects.

## 2. Materials and Methods

### 2.1. Protocol and Registration

The scoping review was conducted using a systematic approach, following the JBI methodology for scoping reviews [28], and is reported in accordance with the Preferred Reporting Items for Systematic reviews and Meta-analyses extension for Scoping Reviews (PRISMA-ScR) Checklist [29] (Supplementary File S1). A preliminary search for previous scoping reviews and systematic reviews on the topic was conducted in Medline via PubMed on 5 December 2021.

### 2.2. Aims and Methodology

The aims and method for this review were prospectively documented in the Open Science Framework Registry (https://osf.io/g483m, accessed on 5 April 2022). The search strategy was developed by two authors (JJ and CH) and a review, consisting of keywords and controlled vocabulary terms, was conducted by a research librarian (Supplementary File S2). A search was conducted across seven included electronic databases (MEDLINE, EMBASE, CINAHL, Cochrane CENTRAL, JBI Evidence Synthesis, Scopus, and Web of Science) and clinical trial registries (ISRCTN registry, clinicaltrials.gov, and World Health Organization International Clinical Trials Registry) on 5 February 2022. Sources that were published in English were included. To provide a comprehensive map of the literature, no restrictions were placed on the publication dates of sources. The sources yielded from the search were imported into the Covidence software [30], where duplicates were removed by the software and confirmed by two researchers in the study. Screening and selection of articles were conducted independently by two authors via Covidence [30] using the study inclusion and exclusion criteria (Table 1). Discrepancies regarding the inclusion of articles were resolved via consensus. In this study, dietary supplements were defined as vitamins, minerals, proteins/amino acids, fatty acids, prebiotics/fiber, probiotics, and plant/herbal extracts, while oral nutrition supplements (ONS) were defined as liquid-based unenriched or unfortified energy-protein formulations. In line with the definition of ‘dietary supplements’ [31], only supplements which were delivered orally were considered. This was extended to include enteral routes to account for people who require tube feeding. While it was recognized that people with metastatic cancers may require alternate routes of delivery (e.g., intravenous or intramuscular), these were not included, as these absorption pathways (and subsequently, required doses) differ from that of oral administration.

Where there was missing information that precluded a decision on the inclusion or exclusion of the article, corresponding authors of the article were emailed to retrieve more information. Data extraction was performed jointly by two authors (JJ and CH) using a data extraction form (Supplementary File S3) that was developed by the team and pilot tested by the same two authors (JJ and CH) prior to use to ensure all relevant results were extracted. Extracted data were subsequently reviewed by another author (RJ). Data extraction included information on the dietary supplements that have been investigated in the target population, as well as their hypothesized effects and proposed mechanisms, and outcome measures that have been assessed. In line with the purpose of scoping reviews outlined in the literature, the present scoping review was intended to map and summarize available evidence, without investigating effectiveness or formulating recommendations for clinical practice [33]. Hence, findings on the actual outcomes of the included studies were not analyzed. Findings of interest to the present scoping review (supplement types, hypothesized effects, proposed mechanisms, and outcome measures used) were narratively synthesized. 

## 3. Results

### 3.1. Search Results

**Figure 1:**
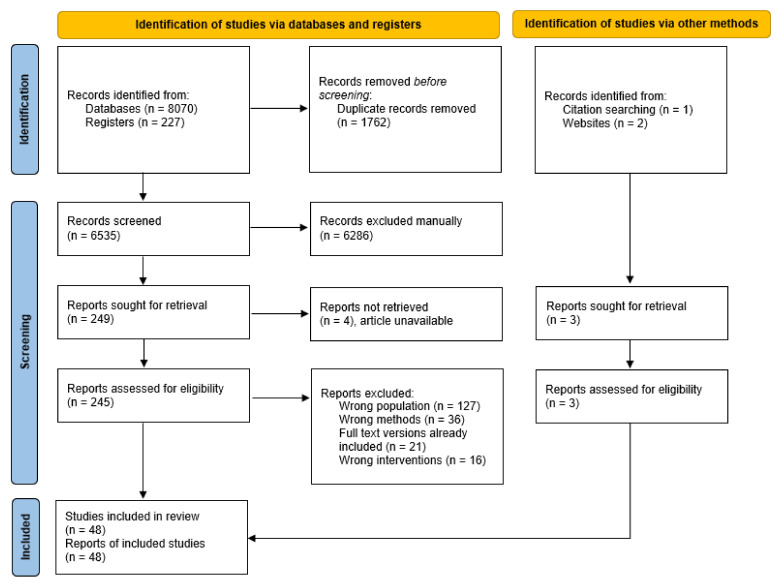
Preferred Reporting Items for Systematic Reviews and Meta-analyses (PRISMA 2020) flow diagram.

The initial search yielded 8070 records from the databases, 227 records from the clinical trial registries, and 3 records from other sources (Figure 1). After removal of duplicates, 6535 records remained, of which 6286 were excluded following title and abstract screening. Of the 248 full-text articles assessed for eligibility, 200 were excluded. A total of 48 articles met the criteria and were included in narrative synthesis.

### 3.2. Study Characteristics

The 48 articles were classified into the following categories: full text peer-reviewed manuscripts (n = 38), conference abstracts (n = 8), and clinical trial registrations (n = 2). Of the full text peer-reviewed manuscripts, the majority reported on randomized controlled trials (RCTs) (n = 18) [34,35,36,37,38,39,40,41,42,43,44,45,46,47,48,49,50,51], followed by quasi-experimental trials (n = 15) [52,53,54,55,56,57,58,59,60,61,62,63,64,65,66], retrospective cohort observational studies (n = 2) [67,68], and case studies (n = 3) [69,70,71]. Conference abstracts reported on RCTs (n = 3) [72,73,74], quasi-experimental trials (n = 4) [75,76,77,78], and a case study (n = 1) [79]. The two clinical trial registrations consisted of one RCT [80], which was terminated as sample size could not be reached, and one quasi-experimental trial [81], which has not commenced recruitment. It was noted that two articles [42,74] may have included the same subset of patients with gynecological cancers, though one of the trials also included additional patients with mixed cancer types [74]. The number of studies and the corresponding range of sample sizes across the different study types, cancer types, and countries are all included in Table 2. 

### 3.3. Types of Dietary Supplements

A variety of dietary supplements were investigated across studies and were grouped into the following categories: vitamins (n = 13), minerals (n = 5), antioxidants (apart from vitamins and minerals) (n = 7), proteins (n = 3), amino acids (n = 14), fatty acids (n = 18), fiber (n = 1), and others (n = 5). Dietary supplements were provided orally in all except three studies [36,67,71], where they were administered enterally. Table 3 shows the types of dietary supplements, cancers, and malnutrition-related conditions in each of the different studies.

### 3.4. Forms and Dosages

#### 3.4.1. Vitamins, Minerals, and Other Antioxidants 

Vitamins included in studies were vitamin A (n = 2) [44,54], B1 (n = 1) [76], B6 (n = 2) [70,76], B9 (n = 4) [46,60,61,68], B12 (n = 1) [60], C (n = 2) [44,54], D (n = 4) [40,59,62,64], and E (n = 3) [39,44,54]. Minerals included were calcium (n = 2) [62,64], iron (n = 2) [68,79], and selenium (n = 1) [75]. Antioxidants (other than vitamins and minerals) included carbocysteine (n = 4) [42,44,54,74], lipoic acid (n = 4) [42,44,54,74], quercetin (n = 2) [44,54], curcumin (n = 2) [73,77], and lycopene (n = 1) [66]. 

The three antioxidant vitamins—Vitamins A, C, and E— were administered concomitantly as part of antioxidant treatments with the same dosages (Vitamin A 30,000 IU daily; Vitamin C 500 mg daily; Vitamin E 400 mg daily) in two studies (an RCT [44] and a quasi-experimental single-group trial [54]) that investigated the efficacy of combined treatments and also included other dietary supplements, such as quercetin, carbocysteine, lipioic acid, eicosapentaenoic acid (EPA), and/or carnitine. In the RCT, all five study arms were given the vitamins [44]. Vitamin E was additionally included in one other RCT, where it was given to the intervention group which received fish oil as the main intervention [39]. 

Vitamins B1 (thiamine) and B6 (pyridoxine) were included in a quasi-experimental trial, in the form of Aminotrofic^®^ sachets, which mainly consisted of amino acids [76]. Vitamin B6 was additionally included in a case study as a replacement therapy in doses of 150 mg daily [70]. Vitamin B9 (folate) was administered in an RCT [46], two quasi-experimental trials [60,61], and an observational study [68], of which all also involved concomitant vitamin B12 administration and pemetrexed therapy. In the RCT, where the main aim was to test the efficacy of different doses of pemetrexed, Vitamin B9 was administered to both study groups via a daily multivitamin containing 500 mg folic acid, along with regular intramuscular vitamin B12 [46]. In one of the quasi-experimental trials, where the aim of the study was to test if the lead-in time for vitamin B supplementation prior to cisplatin-pemetrexed therapy could be shortened, vitamin B9 supplementation in doses of 350–500 µg daily were administered, along with intramuscular vitamin B12 [61]. In the other quasi-experimental trial, where the aim was to evaluate the safety of oral administration of vitamin B12, vitamin B9 (500 µg daily) was administered along with vitamin B12 in patients receiving pemetrexed [60]. In the observational study, vitamin B9 was administered in doses of either 400 µg, 700 µg, or 1000 µg once daily depending on the individual’s baseline total plasma homocysteine level, along with regular oral iron and intramuscular vitamin B12 [68]. The latter study aimed to assess the prevalence of elevated total plasma homocysteine levels at baseline and following pemetrexed treatment, as well as the association between folic acid supplementation and hematological toxicity [68].

One study (a quasi-experimental trial) included vitamin B12 administered orally. The first six participants received 500 µg vitamin B12 daily (along with vitamin B9), while subsequent participants were treated under the updated protocol (following the findings of a study, published then, which showed a lack of efficacy of 1000 µg vitamin B12), where they received 2000 µg daily of vitamin B12 for seven days followed by a dose of 500 µg daily thereafter instead [60]. 

Vitamin D was administered in one RCT [40] and three quasi-experimental trials [59,62,64]. Vitamin D was the sole dietary supplement investigated and was provided in the form of cholecalciferol to participants with vitamin D insufficiency, at a dose of 2000 IU daily in the RCT [40] and 50,000 IU weekly in one of the quasi-experimental trials [59]. In the remaining two quasi-experimental trials, Vitamin D was administered concomitantly with calcium, either in the form of 2000 units ergocalciferol (commercially available liquid vitamin D analogue) daily along with 500 mg calcium daily among participants with insufficient vitamin D [64], or in the form of 0.5 µg 1α-OH vitamin D_3_ (1α-OHD_3_) daily along with 1 g calcium carbonate daily [62].

In an observational study, iron was administered in the form of ferrous sulphate 200 mg twice daily at the initiation of chemotherapy as per clinic protocol, along with oral vitamin B9 and vitamin B12 injection [68]. In a case report, iron was administered in the form of 30 mg sucrosomial iron daily as a supportive intervention to radiation therapy in a patient with sideropenic anemia [79]. Selenium was investigated in only one study (a quasi-experimental trial) and was given in the form of seleno-L-methionine in doses of 2500, 3000, or 4000 µg twice daily for 14 days followed by once daily, in combination with axitinib [75].

In terms of antioxidants other than vitamins and minerals, carbocysteine and lipoic acid were administered concomitantly in three RCTs [42,44,74] and one quasi-experimental trial [54]. A combination of carbocysteine and lipoic acid was administered in two RCTs (that likely shared overlapping participants) as part of an antioxidant treatment (dosage specified as 2.7 g carbocysteine daily and 600 mg lipoic acid daily in one of the RCTs) along with dietary supplement carnitine [42,74]. In the remaining RCT and quasi-experimental trial, carbocysteine (2.7 g daily) and lipoic acid (300 mg daily) were administered in combination with quercetin, as well as EPA and the antioxidant vitamins A, C, E [44,54], and either with or without additional carnitine in the RCT [44]. 

Curcumin was investigated as the sole dietary supplement in a quasi-experimental trial in doses of 2 g daily (equivalent to 400 mg daily of active curcuminoids extract) [77]. In an RCT, curcumin was administered as part of a combined treatment including other dietary supplements (carnitine and lactoferrin) at a dose of 4 g daily [73]. Lycopene was investigated in only one study (quasi-experimental trial), where it was given at a dose of 30 mg daily in people receiving concomitant docetaxel therapy [66]. 

#### 3.4.2. Proteins and Amino Acids 

Protein supplements were investigated in three RCTs. Types of protein supplements included in studies were whey protein isolate (n = 1) [38] and lactoferrin (n = 2) [43,73]. Whey protein isolate was administered in the form of two sachets of cysteine-rich lipid- and lactose-free cow milk whey protein (Prother^®^) consisting of 20 g protein [38]. Lactoferrin was administered as two tablets daily (equivalent to 200 mg daily), along with recombinant human erythropoietin, to people with anemia [43]. In another RCT, the same dose of lactoferrin (200 mg daily) was administered along with carnitine and curcumin, as part of a combined treatment, in people with cancer-related anemia and cachexia [73]. 

For amino acids, carnitine, an amino acid derivative, was the most investigated and was included in seven studies, including five RCTs [41,42,44,73,74] and two quasi-experimental trials [53,56]. It was the sole dietary supplement investigated in two studies [41,53] and part of a combined treatment with other dietary supplements (EPA, branched chain amino acids, coenzyme Q10, lipoic acid, carbocysteine, curcumin, lactoferrin, quercetin, and/or vitamins A, C, and E) in five studies [42,44,56,73,74]. Carnitine was given as L-carnitine and in doses of 50 mg, 2 g, 4 g, or 6 g daily in included studies.

Arginine was the second most commonly investigated amino acid. Arginine was the main dietary supplement investigated in an RCT where it was administered in the form of a specially formulated enteral formula and replaced 41% of casein [36], as well as in an observational study where it was given in the form of an immunonutrition enteral formula (Impact^®^) containing 12.5 g/L L-arginine, dietary nucleotides, EPA, and docosahexaenoic acid (DHA) [67]. Arginine was investigated as part of a combined treatment with glutamine (amino acid) and β-hydroxyl β-methyl butyrate (HMB) (amino acid metabolite) in three studies (two RCTs [35,45] and one quasi-experimental trial [57]), which were also the only studies where glutamine or HMB were included. Daily doses were in the following ranges: 14–28 g arginine, 14–28 g glutamine, and 2.4–6 g HMB daily [35,45,57]. 

Branched chain amino acids (BCAA) were investigated in a quasi-experimental trial, where they were administered in the form of an enriched ONS (Inner Power^®^), which consists of 2500 mg BCAA per pack, along with coenzyme Q10 and carnitine, with one pack given daily [56]. Two studies, an RCT [72] and a quasi-experimental trial [76], included all the essential amino acids. In the RCT, a 4 g essential amino acid powder was the sole intervention and was given thrice daily (equivalent to 12 g amino acids daily) [72]. In the quasi-experimental trial, essential amino acids were given in the form of two sachets of Aminotrific^®^ supplement, which also consisted of vitamins B1 and B6 [76]. 

#### 3.4.3. Fatty Acids

Omega-3 fatty acids, EPA and DHA, were the most commonly investigated dietary supplement overall (n = 18), and were studied in nine RCTs [37,39,44,47,48,49,50,51,80], six quasi-experimental trials [54,55,58,63,65,78], one observational study [67], and two case studies [69,71]. Omega-3 fatty acids were administered in the form of free EPA acids [65], purified EPA + DHA capsules [69], krill oil capsules [78], fish oil capsules [37,39,47,55], fish oil liquid [55], marine phospholipids [63], or in fortified ONS [44,48,49,50,51,54,58,67,71,80]. Marine phospholipids and krill oil consisted of omega-3 fatty acids that were bound to phospholipids, which were suggested by study authors to have a different uptake and metabolism from those bound to triacylglycerols (such as those in fish oil) [63,78]. With the exception of one study where omega-3 was administered enterally [71], omega-3 was given orally for all. Reported doses ranged from 1.1 g to 6 g EPA daily and 0.2 to 2.7 g DHA daily [37,39,44,47,49,50,51,54,55,58,65,69,71]. Omega-3 fatty acids were the only dietary supplement included in 13 studies [37,47,48,49,50,51,55,58,63,65,71,78,80], while additional dietary supplements (e.g., carnitine, arginine, dietary nucleotides, fiber, quercetin, lipoic acid, carbocysteine, and vitamins A, C, and E) were included in five studies [39,44,54,67,69]. Although DHA was not explicitly mentioned in some studies, it was present along with EPA by virtue of the forms in which EPA was administered [44,48,50,51,55,71,80].

#### 3.4.4. Fiber 

Fiber was only included in one case study where omega-3 fatty acids were the main dietary supplement of interest [69]. The participant consumed one serving of fortified ONS (Forticare Nutricia) daily, which contains both EPA and fiber and provides 2.6 g fiber daily. 

#### 3.4.5. Others

Other dietary supplements that were investigated were β-hydroxybutyrate (BHB) [81], coenzyme Q10 [56], muscadine grape extract [52], dietary nucleotides [67], and royal jelly [34]. The supplement BHB was included in a quasi-experimental trial that has not started recruitment yet and will be administered in the form of liquid ketone supplement, two tablespoons three times daily (providing 1 g/kg body weight daily of BHB) [81]. Coenzyme Q10 was administered in a quasi-experimental trial in the form of one pack of enriched ONS (Inner Power^®^ which contains BCAA, carnitine, and 30 mg coenzyme Q10 per pack) daily [56]. Muscadine grape extract was investigated in a quasi-experimental trial in the form of capsules that were taken twice daily (each capsule containing ~160 mg phenolics) in five dose levels of 320 to 1600 mg total phenolics [52]. Royal jelly was administered in the form of 800 mg capsules three times daily (equivalent to 2400 mg daily), as per figures published in the erratum [82]. 

### 3.5. Concomitant Interventions

Of the 48 studies, single dietary supplements were administered on their own, without concomitant interventions, in 19 studies (e.g., omega-3 only) [34,38,40,41,47,52,55,59,62,63,64,65,66,70,72,75,77,78,79]. The remaining 29 studies had single dietary supplements administered with other dietary supplements (e.g., omega-3 and arginine), ONS, counseling, exercise, and/or non-cancer specific drugs. Non-cancer specific drugs prescribed concomitantly in those studies were celecoxib [37,42,50,54,73,74], medroxyprogesterone acetate or megestrol acetate [42,44,54,74], thalidomide [44], or recombinant human erythropoietin [43].

In studies with concomitant interventions, dietary supplements were administered with ONS in 12 studies and were used to fortify/enrich ONS in some studies. Oral nutritional supplements were the only other intervention in three studies [36,49,51], while different types of dietary supplements were used as a combination (i.e., omega-3 and arginine) in addition to ONS in two studies [67,71]. In the remaining studies with ONS, dietary supplements were also used in conjunction with nutritional counseling [58,80], prescribed diet [48], drug [37], or a combination of drug, nutritional counseling, and exercise (home-based aerobic and resistance training) [50], or were part of a combination of dietary supplements and drugs [44,54]. In the 17 studies with concomitant interventions but without ONS, dietary supplements were delivered concurrently with drugs [43] or nutritional counseling [38], or as part of a combination with other dietary supplements [35,39,45,46,60,61,68,76] that were accompanied by drugs [42,73,74], or in-person structured nutrition, exercise, and symptom advice via the Macmillan Durham Cachexia Pack [57], or in-person individualized nutritional and exercise counseling along with prescription of individualized home-based resistance training [56].

### 3.6. Malnutrition-Related Conditions

The malnutrition-related conditions investigated in the included studies included cachexia (n = 14) [37,44,50,51,56,57,63,70,71,72,73,74,77,78], weight loss (n = 10) [35,41,42,45,47,48,54,55,56,65], malnutrition (n = 6) [36,38,39,48,58,67], vitamin D deficiency (n = 3) [40,59,64], anorexia (n = 2) [37,47] vitamin B6 deficiency (n = 1) [70], muscle depletion (n = 1) [56], and cancer-related anemia (n = 1) [43]. The specific malnutrition-related conditions were not specified in other studies (n = 15) [34,46,49,52,53,60,61,62,66,68,69,75,76,80,81]. Cachexia was most commonly investigated, and where reported, definitions were variable and included the following: >2% weight loss in patients with BMI <20 kg/m^2^ [56], >5% weight loss according to ideal or pre-illness body weight in the past three months [44], >5% weight loss in the past six months [56,73], >5% weight loss since first diagnosis [63], >10% weight loss [37], or the presence of muscle depletion [56]. No study investigated frailty or sarcopenia (both in accordance with published definitions), and only one study included patients with muscle depletion [56], defined by lumbar skeletal muscle index cut-offs [83]. 

### 3.7. Cancer Types

**Figure 2:**
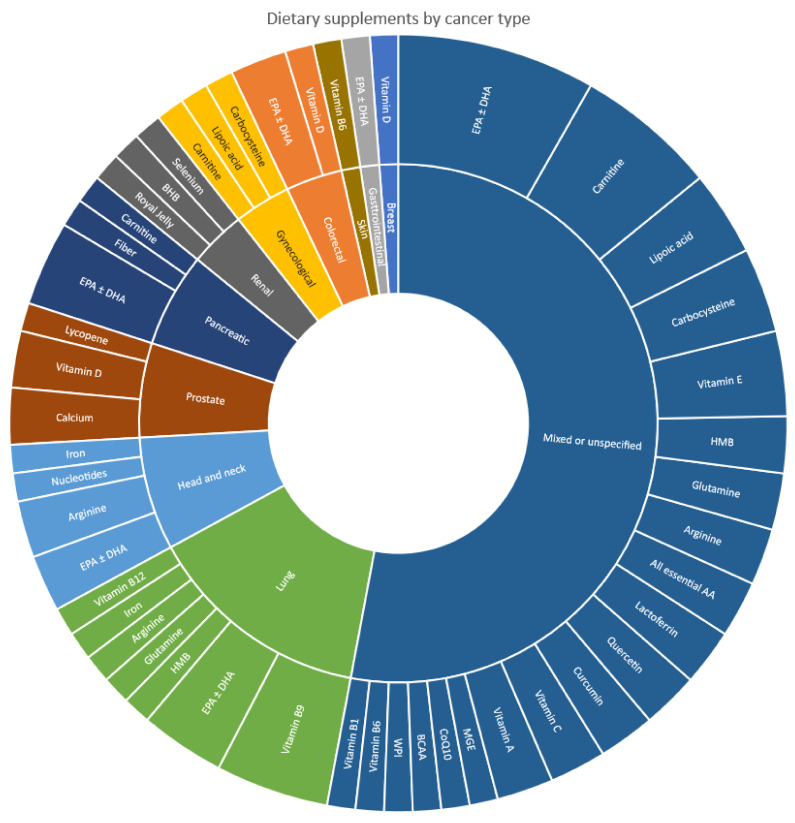
Types of dietary supplements included in studies, according to cancer type.

Dietary supplements that were investigated in the included studies varied across different cancer types, as illustrated in Figure 2. Omega-3 fatty acids (EPA and/or DHA) showed the most consistency, where they were among the most ubiquitous dietary supplements for mixed or unspecified cancer types (n = 7) [39,44,47,50,54,63,78], as well as for the individual cancer types—lung (n = 3) [37,48,55], head and neck (n = 2) [67,71], pancreatic (n = 3) [51,65,69], and colorectal cancers (n = 2) [58,80].

In terms of individual cancer types, and beyond omega-3, the predominant dietary supplements investigated were vitamin B9 in lung cancer (n = 4) [46,60,61,68], arginine in head and neck cancer (n = 2) [36,67], and vitamin D plus calcium in prostate cancer (n = 2) [62,64]. All remaining dietary supplements investigated in individual cancer types were present only in one study in each of the cancer types. For mixed or unspecified cancer types, carnitine (n = 5) [44,53,56,73,74] was most commonly prescribed after omega-3, followed by vitamin E (n = 3) [39,44,54], carbocysteine (n = 3) [44,54,74], and lipoic acid (n = 3) [44,54,74]. The remaining dietary supplements investigated in mixed or unspecified cancer types were only present in one or two studies each. 

### 3.8. Proposed Effects, Mechanisms/Rationale, and Outcome Measures 

As shown in Table 4, dietary supplements were hypothesized to improve malnutrition-related conditions in a majority of the studies [35,37,38,41,42,43,44,45,47,48,49,50,51,54,57,59,63,64,65,71,72,73,74,76,77,78,79]. In some studies, the hypothesized main effects of dietary supplements were related to benefits that were not directly relevant to malnutrition-related conditions. These were improvements in survival [40], symptoms of cancer metastasis (e.g., pain) [64], antitumor efficacy of chemotherapy [66], and treatment response [75]; counteracting adverse effects of chemotherapy, (e.g., anti-folate effects of pemetrexed) [46,60,61,68], bone loss from complete androgenic blockade use [62], muscle and joint pain from estrogen blockade use [59], and inflammation; oxidative stress immune system dysfunction induced by tyrosine kinase inhibitors [34]; and counteracting adverse effects of other dietary supplements that are concomitantly administered (such as compensating for the oxidative effect of omega-3 supplements) [39].

The intended attributes of dietary supplements included antioxidant [39,42,44,49,52,54,63,66,71,74,77], anti-inflammatory [34,37,41,43,44,48,49,51,52,63,65,67,71,77], anti-cancer [36,40,48,64,65,66], and immunomodulatory [34,36,38,45,49,63,65,67,71] properties; ability to reduce infections [43,67], facilitate wound healing [35,67], improve physical function [56], improve muscle strength [64], mitigate muscle loss and reduce muscle damage [35,45,57], improve muscle trophism [38,76], and regulate protein synthesis or turnover [35,57]; and having a role in energy and amino acid metabolism [42,44,53,73].

### 3.9. Tools Used in Outcome Measurements 

The tools that were used to assess each of the different outcome measures, where specified, are presented in Table 5. 

## 4. Discussion

This scoping review summarized the types of dietary supplements used in studies involving metastatic cancer patients with malnutrition-related conditions. Thirty-one supplements were identified, which varied across different cancer types. Dietary supplements were investigated as part of combined treatments in most of the studies, where they could be administered as single (i.e., omega-3) or multiple types (i.e., omega-3 + arginine), or along with ONS, other dietary supplements, counseling, exercise, and/or drugs. Omega-3 and L-carnitine were the top two most predominantly investigated supplements—omega-3 for its anti-inflammatory [37,44,49,51,63,71], antioxidant [49,63,71], and immunomodulatory [49,63,65,71] properties, and carnitine for its suggested benefits in the modulation of inflammatory response mechanisms that have been associated with cancer cachexia [41] and role in metabolism [42,44,53,73]. To combat oxidative stress and manage the complexity of cancer-related malnutrition, multimodal treatments were considered necessary to reduce proinflammatory cytokines [44,54]. Metastasis itself is a complex challenge that necessitates multimodal therapeutic agents for effective inhibition [2] and managing its associated syndromes. While multimodal interventions may confer benefits over single interventions, it is challenging to identify the individual contribution of dietary supplements to any beneficial effects seen. Hence, it might be worthwhile investigating this with multi-arm RCTs, including both single and combined interventions which are compared to controls in future studies. 

Overall, the included studies consistently reported positive effects for multimodal treatments as well as omega-3 supplements. Evidence for vitamins, minerals, and amino acids was less consistent. While antioxidants and other dietary supplements were reported by studies to exert positive effects, the number of studies that they have been included in were scant. As the present scoping review was, however, not designed to investigate effectiveness, critical appraisal and synthesis of outcome findings of the included studies were not carried out, and recommendations regarding their use cannot be made within the scope of this review. 

Cancer-related malnutrition is a complex condition attributable to the imbalance of in vivo redox systems (including antioxidant enzymes and antioxidants) and upregulation of proinflammatory cytokines [44,54]. Omega-3 has the ability to inhibit the production of proinflammatory cytokines [48,49,63,65,71], and thus holds promise in its potential to manage this syndrome [44,51,63,65]. Two previous systematic reviews (one in adults with cancer undergoing chemotherapy and/or radiotherapy [84] and the other in adults with cancer cachexia who were not undergoing cancer treatment during the study period [85]) indicated beneficial effects (e.g., improvements in body composition, weight, appetite, QoL) of omega-3 fatty acid supplements (EPA; DHA). However, a systematic review in patients with advanced cancer (which included locally recurrent cancers in definition) [86] did not find sufficient evidence to support the superiority of omega-3 fatty acid supplements (specifically EPA) over placebo. As the Cochrane review was conducted over 15 years ago, it may be useful to conduct an updated review focusing on patients with metastatic cancer, as more primary studies become available. This can also help identify the effectiveness of dietary supplements in managing cancer-related malnutrition and address the lack of Level 1 evidence in this population. 

Overall, more primary studies are warranted for the dietary supplements elucidated in this review. For example, carnitine was investigated in seven studies, of which only two studies [41,53] investigated it as the sole intervention. Additionally, the present review has mapped the types of dietary supplements to their proposed usefulness for particular cancer types (i.e., vitamin D for prostate cancer). This may provide some indication to researchers regarding the potentially efficacious dietary supplements that can be investigated in studies for specific cancer types. 

The strengths of this review include its methodological rigor in line with standards and guidance for scoping review conduct and reporting, the dual approach to screening and extraction to reduce error, and the comprehensive search strategy. Additionally, a wide scope of all available evidence at varying levels on the evidence hierarchy was included. The limitations of the present review include the exclusion of studies where dietary factors (e.g., vitamins, minerals, fatty acids) were administered via the intramuscular and intravenous routes. These were excluded as they were technically not dietary supplements [87]; however, they might have otherwise provided additional useful information. Additionally, while efforts were made to contact study authors where missing information precludes the inclusion of a paper, only a small number of replies (5/38) were received. Lastly, as none of the included studies specified the inclusion of children, sarcopenic, or frail populations, the present review is unable to provide information on these population groups. 

### Implications for Research

With the identified dietary supplements and their noteworthy mechanisms and rationale for use in patients with metastatic cancers, particularly for omega-3, vitamin D, and amino acids (arginine, carnitine, glutamine, and HMB), future research in this area is required to assess efficacy on patient outcomes. Future studies should consider conducting fully powered RCTs to increase the reliability of the results. With most of the existing studies having been conducted among mixed or unspecified cancer populations, it would be worthwhile to investigate the efficacy of dietary supplements in specific cancer types, particularly in cancers where malnutrition-related issues are more prevalent (e.g., head and neck cancers). As the forms of dietary supplements were considered to make a difference in some cases (i.e., purported superiority of phospholipids-bound omega-3 over triacylglycerols-bound), researchers may consider the merit of the different forms of dietary supplements when designing future studies. There is also a need to report malnutrition with validated nutritional assessment tools (e.g., PG-SGA) in specific cancer cohorts (e.g., breast, lung, brain). 

## 5. Conclusions

Dietary supplements investigated in studies conducted among patients with metastatic cancers were multifarious and differed across cancer types. With plausible effects and mechanisms proposed in relation to their role in managing malnutrition-related conditions in this population group, future studies assessing the efficacy of dietary supplements on patient outcomes are needed. As primary trials are still lacking for most of the dietary supplements, future RCTs can be considered, along with a consideration of the forms of dietary supplements to be tested, concomitant interventions to be employed (if any), and the relevance of dietary supplements to the specific cancer type of interest.

## Figures and Tables

**Table 1:** List of inclusion and exclusion criteria.

Inclusion Criteria	Additional Operational Information
Human studies	-
Primary, quantitative studies, or systematic reviews	Systematic reviews are defined as reviews with a comprehensive search strategy, methods section, and critical appraisal of included studies
Studies investigating the effects of dietary supplements (vitamins, minerals, proteins or amino acids, fatty acids, prebiotics or fiber, probiotics, and plant or herbal extracts) whether in isolation or in combination with other dietary supplements or interventions	This includes studies where dietary supplements were not the intervention of interest or all study groups received the same dietary supplements, as intra-group comparisons may have been made, which can provide information on the effects of dietary supplements.
Studies conducted among people with metastatic cancer (defined as Stage IV)	This is further defined by the following cut-off values: for primary trials, at least 50% metastatic; for systematic reviews, at least 50% included papers conducted in solely metastatic populations; for studies yet to be completed, only those that set out to recruit solely patients with metastatic cancers.
Studies including people with malnutrition cachexia or anorexia, sarcopenia, frailty, or weight loss	Studies that consisted of a subset of patients (any proportion) with these conditions were included. Nutrient deficiencies were also considered a form of malnutrition. If the nutrition status of study participants was not specified, articles were considered to meet the inclusion criteria regarding malnutrition-related conditions as long as they meet the earlier criteria of metastatic cancer, as it has been established in the literature that malnutrition is prevalent in people with advanced cancer [32].
**Exclusion Criteria**	
Animal or laboratory studies	
Qualitative studies or narrative/literature reviews	
Studies investigating the effects of drugs or traditional medicine (e.g., Chinese herbal therapies)	
Supplements administered via intravenous or intramuscular routes (e.g., intravenous ascorbic acid infusion)	
Studies investigating the effect of oral nutritional supplements alone, which have not been enhanced with dietary supplement(s) of interest (e.g., unfortified standard formulations of commercial milk-based supplements, such as Ensure^®^)	

**Table 2:** Number of completed studies (excluding one clinical trial registration which has not started recruitment) and number of participants included, according to study type, cancer type, and country.

Study Type	Number of Studies	Total Participants Range
**Full-text article**		
Randomized controlled trial	18	22–472
Quasi-experimental trial	15	12–144
Retrospective cohort observational	2	111–135
Case study	3	1
**Conference abstracts**		
Randomized controlled trial	3	50–127
Quasi-experimental trial	4	12–36
Case study	1	1
**Clinical trial registrations**		
Randomized controlled trial	1	13
**Cancer Type (Primary)**		
Breast	1	11
Colorectal	3	13–72
Gastrointestinal	1	128
Gynecological	1	104
Head and neck	4	1–135
Lung	8	22–225
Pancreatic	4	1–72
Prostate	3	13–51
Renal	2	12–33
Skin	1	1
Mixed or not specified	19	12–472
**Country**		
Argentina	1	22
Australia	1	23
Canada	2	23–144
Croatia	1	72
Germany	3	31–72
Greece	1	60
India	1	111
Italy	12	1–332
Japan	8	1–225
Mexico	1	92
Netherlands	1	32
Poland	1	1–51
Portugal	1	1
Scotland	1	26
Spain	2	13–135
United States	8	1–472
United Kingdom	2	38–46

**Table 3:** Types of dietary supplements, cancers, and malnutrition-related conditions included in each of the studies.

Author/ Year Trial No./ Clinical Trial Phase	Cancer	Conditions	Vitamins								Minerals			Antioxidants					Proteins		Amino Acids						FA	Fr	Others				
			Vitamin A	Vitamin B1	Vitamin B6	Vitamin B9	Vitamin B12	Vitamin C	Vitamin D	Vitamin E	Calcium	Iron	Selenium	Carbocysteine	Curcumin	Lipoic acid	Lycopene	Quercetin	Lactoferrin	Whey protein isolate	Essential amino acids	Arginine	BCAA	Carnitine	Glutamine	HMB	EPA ± DHA	Fiber	BHB	Coenzyme Q10	Muscadine Grape Extract	Nucleotides	Royal Jelly
**Full-Text Article**																																	
**RCT**																																	
Araki 2019 [34] UMIN000020152 Phase NS	Renal	NS																															**X**
Berk 2008 [35] Trial no. NS Phase III	Mixed	Weight loss																				**X**			**X**	**X**							
Buijs 2010 [36] Trial no. and phase NS	HN	Malnutrition																				**X**											
Cerchietti 2007 [37] Trial no. and phase NS	Lung	Cachexia, anorexia																									**X**						
Cereda 2019 [38] NCT02065726 Phase NS	Mixed	Malnutrition																		**X**													
Gogos 1998 [39] Trial no. and phase NS	Mixed	Malnutrition								**X**																	**X**						
Golubić 2018 [40] Trial no. and phase NS	COL	Vitamin D insufficiency							**X**																								
Kraft 2012 [41] NCT01330823 Phase NS	PanC	Weight loss																						**X**									
Maccio 2012 [42] Trial no. NS Phase III	GYN	Weight loss												**X**		**X**								**X**									
Maccio 2010 [43] Trial no. and phase NS	Mixed	Anemia																	**X**														
Mantovani 2010 [44] Trial no. NS Phase III	Mixed	Cachexia	**X**					**X**		**X**				**X**		**X**		**X**						**X**			**X**						
May 2002 [45] Trial no. and phase NS	Mixed	Weight loss																				**X**			**X**	**X**							
Ohe 2008 [46] Trial no. NS Phase II	Lung	NS				**X**																											
Pratt 2002 [47] Trial no. and phase NS	Mixed	Anorexia, weight loss																									**X**						
Sanchez-Lara 2014 [48] NCT01048970 Phase NS	Lung	Malnutrition, weight loss																									**X**						
Shirai 2017 [49] Trial no. and phase NS	GI	NS																									**X**						
Solheim 2017 [50] NCT01419145 Phase II	Mixed	Cachexia																									**X**						
Ueno 2022 [51] UMIN000003658 Phase II	PanC	Cachexia																									**X**						
**Quasi-experimental trial**																																	
Bitting 2021 [52] NCT02583269 Phase I	Mixed	NS																													**X**		
Gramignano 2006 [53] Trial no. and phase NS	Mixed	NS																						**X**									
Mantovani 2006 [54] Trial no. NS Phase II	Mixed	Weight loss	**X**					**X**		**X**				**X**		**X**		**X**									**X**						
Murphy 2011 [55] Trial no. NS Phase I	Lung	Weight loss																									**X**						
Naito 2019 [56] UMIN000023207 Phase NS	Mixed	Cachexia, muscle/weight loss																					**X**	**X**						**X**			
Pascoe 2021 [57] ISRCTN39911673 Phase II	Lung	Cachexia																				**X**			**X**	**X**							
Read 2007 [58] Trial no. NS Phase II	COL	Malnutrition																									**X**						
Sheean 2021 [59] NCT02186015 Phase I	Breast	Vitamin D insufficiency							**X**																								
Takagi 2016 [60] UMIN000003180 Phase II	Lung	NS				**X**	**X**																										
Takagi 2014 [61] UMIN000006546 Phase II	Lung	NS				**X**																											
Talalaj 2005 [62] Trial no. and phase NS	ProST	NS							**X**		**X**																						
Taylor 2010 [63] Trial no. and phase NS	Mixed	Cachexia																									**X**						
Van Veldhuizen 2000 [64] Trial no. NS Phase II	Prostate	Vitamin D insufficiency							**X**		**X**																						
Wigmore 2000 [65] Trial no. NS Phase I/II	PanC	Weight loss																									**X**						
Zhuang 2021 [66] NCT01882985 Phase II	ProST	NS															**X**																
**Retrospective cohort observational**																																	
Barajas-Galindo 2020 [67] Trial no. and phase NS	HN	Malnutrition																				**X**					**X**					**X**	
Singh 2017 [68] Trial no. and phase NS	Lung	NS				**X**						**X**																					
**Case study**																																	
Ramalho 2017 [69] Trial no. and phase N/A	PanC	NS																									**X**	**X**					
Rauf 2011 [70] Trial no. and phase N/A	Skin	Vitamin B6 deficiency, cachexia			**X**																												
Yoshii 2014 [71] Trial no. and phase NS	HN	Cachexia																									**X**						
**Conference Abstract**																																	
**RCT**																																	
Haehling 2017 [72] Trial no. and phase NS	Mixed	Cachexia																			**X**												
Madeddu 2014 [73] Trial no. and phase NS	NS	Cachexia													**X**				**X**					**X**									
Madeddu 2012 [74] Trial no. NS Phase III	Mixed	Cachexia												**X**		**X**								**X**									
**Quasi-experimental trial**																																	
Garje 2019 [75] NCT02535533 Phase I	Renal	NS											**X**																				
Lugini 2013 [76] Trial no. NS Phase II	NS	NS		**X**	**X**																**X**												
Mantovani 2012 [77] Trial no. and phase NS	Mixed	Cachexia													**X**																		
Serpe 2012 [78] Trial no. and phase NS	Mixed	Cachexia																									**X**						
**Case Report**																																	
Ricottone 2017 [79] Trial no. and phase N/A	HN	Anemia										**X**																					
**Clinical Trial Registration**																																	
**RCT**																																	
NCT00398333 [80] Phase IV	COL	NS																									**X**						
**Quasi-experimental trial**																																	
NCT05119010 [81] Phase NS	Renal	NS																											**X**				

NS: not specified; BCAA: branched chain amino acids; HMB: β-hydroxyl β-methyl butyrate; EPA: eicosapentaenoic acid; DHA: docosahexaenoic acid; BHB: β-hydroxybutyrate; HN: head and neck; FA: fatty acids; Fr: Fiber; COL: Colorectal; GYN: Gynecological; GI: Gastrointestinal; PanC: Pancreatic; ProST: Prostate.

**Table 4:** Hypothesized effects of dietary supplements, mechanisms/rationale, and outcomes measures assessed.

Dietary Supplement	Hypothesized Effects	Proposed Mechanisms or Rationale	Outcome Measures in Studies Where the Dietary Supplement Was Included
Vitamins, minerals, and other antioxidants			
Vitamin A	Functions as an antioxidant agent to improve CACS as part of an integrated treatment [44,54].	Reduces proinflammatory cytokines to improve oxidative stress and CACS symptoms, as part of a combination of antioxidants, ONS, and drugs [44,54].	Weight; LBM; grip strength; REE; leptin, proinflammatory cytokines, antioxidant enzymes, and ROS levels; appetite; fatigue; physical activity level; performance status; QoL; prognostic score.
Vitamin B1	Contributes to prevention of anorexia and cachexia along with Vitamin B6 and amino acids during chemotherapy [76].	Useful in muscle trophism, along with Vitamin B6 and amino acids [76].	Nutritional status, clinical status, QoL, adherence to chemotherapy.
Vitamin B6	Oral replacement therapy corrects neutropenia that stems from Vitamin B6 deficiency [70]. Contributes to prevention of anorexia and cachexia along with Vitamin B1 and amino acids during chemotherapy [76].	Oral replacement therapy corrects Vitamin B6 deficiency and its associated neutropenia [70]. Useful in muscle trophism, along with Vitamin B1 and amino acids [76].	Nutritional status; vitamin B6 and neutrophil levels; clinical status; QoL, regression of cervical adenopathy; adherence to chemotherapy.
Vitamin B9	Reduces adverse events from pemetrexed therapy [46,60,61,68].	Reduces toxicity to pemetrexed (an antifolate) when used with Vitamin B12 [46,60,61,68]. Vitamin B9 dosing, however, needs to be optimal to prevent interference with pemetrexed effectiveness [68].	Total plasma homocysteine levels, QoL, relative dose intensity, tumor response, survival, adverse events (including neutropenia grade and other toxicities).
Vitamin B12	Oral vitamin B12 is an alternative to intramuscular vitamin B12 for purposes of reducing pemetrexed-associated adverse events, when used along with Vitamin B9 [60].	Oral administration of vitamin B12 is capable of correcting vitamin B12 deficiency and is, thus, an alternative to intramuscular injection [60].	Total plasma homocysteine levels, tumor response, survival, adverse events (including neutropenia grade and other toxicities).
Vitamin C	Functions as an antioxidant agent to improve CACS as part of an integrated treatment [44,54].	Reduces proinflammatory cytokines to improve oxidative stress and CACS symptoms, as part of a combination of antioxidants, ONS, and drugs [44,54].	Weight; LBM; grip strength; REE; leptin, proinflammatory cytokines, antioxidant enzymes and ROS levels; appetite; fatigue; performance status; physical activity; QoL; prognostic score.
Vitamin D	Improves survival in metastatic colorectal cancer [40]. Replacement therapy improves muscle strength and pain associated with prostate cancer bone metastasis [64]. 1α-OHD3 along with calcium supplementation prevents bone mass loss in men with prostate cancer treated with complete androgenic blockade [62]. Improves symptom burden in women with estrogen receptive–positive metastatic breast cancer [59].	Exerts anti-cancer effects when converted to calcitriol in the body, by regulating cancer-related genes [40]. Inhibits growth of prostate cancer cells [64]. 1α-OH vitamin D3, along with calcium, prevents bone mass loss [62]. Supplementation improves vitamin D insufficiency to reduce muscle and joint pain that stem from estrogen blockade use and low serum 25-hydroxyvitamin D (25[OH]D) levels [59].	Anthropometry, bone mineral density, muscle strength, grip strength, 25(OH)D levels, calcium phosphatase and alkaline phosphatase levels, prostate specific antigen levels, CRP levels, proinflammatory cytokines levels, symptom burden, pain, QoL, survival.
Vitamin E	Functions as an antioxidant agent to improve CACS as part of an integrated treatment [44,54]. Minimizes side effects of omega-3 supplementation [39].	Reduces proinflammatory cytokines to improve oxidative stress and CACS symptoms, as part of a combination of antioxidants, ONS, and drugs [44,54]. Compensates for the oxidative effect of omega-3 [39].	Weight; LBM; grip strength; REE; leptin, albumin, transferrin, proinflammatory cytokines, T-cell subsets, antioxidant enzymes, and ROS levels; appetite; fatigue; performance status; physical activity; QoL; prognostic score; survival.
Calcium	Prevents bone mass loss in men treated with complete androgenic blockade, along with 1α-OHD3 [62].	Calcium, along with 1α-OH vitamin D3, prevents bone mass loss, which people with advanced prostate cancer receiving complete androgenic blockade are more susceptible to [62].	Bone mineral density; muscle strength; 25(OH)D, calcium phosphatase, alkaline phosphatase, and prostate specific anti-gen levels; pain.
Iron	Improves sideropenic anemia when administered in the form of sucrosomial iron, as a supportive therapy alongside radiation therapy [79].	As radiation therapy can lead to side-effects such as mucositis and dysphagia resulting in malnutrition and subsequent onset of sideropenic anemia, sucrosomial iron improves sideropenic anemia when given concomitantly with radiation therapy [79].	Weight; hemoglobin, mean corpuscular, mean corpuscular hemoglobin, and total plasma homocysteine levels; mucositis; tumor response; toxicity.
Selenium	Improves axitinib therapy response when administered in the form of seleno-L-methionine [75].	Seleno-L-methionine stabilizes tumor vasculature and reduces risks of angiogenesis, tumor metastasis and treatment resistance when given in combination with chemotherapeutic and vascular endothelial growth factor–targeted agents [75].	Response, survival, adverse events; toxicity.
Carbocysteine	Functions as an antioxidant agent to improve cancer cachexia symptoms as part of an integrated treatment [42,44,54,74].	Reduces proinflammatory cytokines to improve oxidative stress and CACS symptoms, as part of a combination of antioxidants, ONS, and drugs [44,54]. Important precursor of cell-reduced glutathione and counteracts oxidative stress [42].	Weight; LBM; grip strength; REE; leptin, CRP, proinflammatory cytokines, ROS, and antioxidant enzyme levels; appetite; fatigue; performance status; physical activity level; QoL; prognostic score; adverse events.
Curcumin	Improves nutritional and immunometabolic alterations of cachexia and cancer-related anemia as part of a combined treatment also consisting of L-carnitine, lactoferrin and celecoxib [73]. Exerts antioxidant and anti-inflammatory effects in advanced cancer cachexia [77].	Possesses anti-inflammatory and antioxidant effects to mitigate inflammation and oxidative stress in cachexia [73,77].	LBM; serum iron, ferritin, hepcidin, erythropoietin, CRP, proinflammatory cytokines, ROS, antioxidant enzyme, and total blood antioxidant status levels; appetite; fatigue; anemia.
Lipoic acid	Functions as an antioxidant agent to improve cancer cachexia symptoms as part of an integrated treatment [42,44,54,74].	Reduces proinflammatory cytokines to improve oxidative stress and CACS symptoms as part of a combination of antioxidants, ONS, and drugs [44,54]. Important precursor of cell-reduced glutathione and counteracts oxidative stress [42].	Weight; LBM; grip strength; REE; leptin, CRP, proinflammatory cytokines, ROS, and antioxidant enzyme levels; appetite; fatigue; performance status; physical activity level; QoL; prognostic score; adverse events.
Lycopene	Concomitant administration with docetaxel is an effective treatment for prostate cancer [66].	Possesses antioxidant properties and has chemo preventive effects in prostate cancer; inhibits antiapoptotic protein, and improves antitumor efficacy of docetaxel [66].	Prostate specific antigen response, survival, adverse events.
Quercetin	Functions as an antioxidant agent to improve CACS as part of an integrated treatment [44,54].	Reduces proinflammatory cytokines to improve oxidative stress and CACS symptoms when used in a combination of antioxidants, ONS, and drugs [44,54].	Weight; LBM; grip strength, REE; leptin, proinflammatory cytokines, antioxidant enzymes, and ROS levels; appetite; fatigue; performance status; physical activity level; QoL; prognostic score.
**Proteins and amino acids**			
Lactoferrin	Improves nutritional and immunometabolic alterations of cachexia and cancer-related anemia as part of a combined treatment with L-carnitine, curcumin and celecoxib [73]. An alternative to intravenous iron supplementation when combined with recombinant human erythropoietin, in the treatment of anemia in advanced cancer during chemotherapy [43].	Key in host defense against infection and excessive inflammation [43]. As patients with cancer anemia may have low or normal serum iron levels, yet increased ferritin levels and rich bone marrow iron reserves, it is suggested that cancer anemia is associated with flaws in iron use rather than iron shortage [43]. Lactoferrin, which is involved in iron transport mechanisms, can thus treat this form of iron-related anemia [43,73].	LBM; hematopoietic response; erythrocyte sedimentation rate; hemoglobin, iron, ferritin, hepcidin, erythropoietin, CRP, proinflammatory cytokine, ROS, and antioxidant enzyme levels; appetite; fatigue; anemia; adverse events.
Whey protein isolate	Improves nutritional status of malnourished advanced cancer patients receiving chemotherapy [38].	Possesses immune-enhancing factors and contains cysteine (a limiting amino acid in the glutathione production), where glutathione protects cells from free radicals and carcinogens. Induces more muscle protein synthesis, being more rapidly digested than other protein sources [38].	Weight, phase angle, fat-free mass index, grip strength, protein calorie intake, QoL, chemotherapy toxicity.
All essential amino acids	Counter wasting processes associated with cancer cachexia [72]. Prevents anorexia and cachexia in cancer during chemotherapy, along with vitamins B1 and B6 [76].	Useful in muscle trophism, along with vitamins B1 and B6 [76].	Weight, body composition, nutritional status, clinical status, muscle strength, exercise capacity, QoL, adherence to chemotherapy.
Arginine	Prevents cancer recurrence following surgical removal of malignant tumors, especially when administered perioperatively [36]. Improves postoperative recovery as part of an immunonutrition enteral formula in head and neck cancer [67]. Prevents LBM loss and reverses cancer cachexia, as part of a mixture with HMB and glutamine [35,45]. Delays cachexia onset in advanced lung cancer [57].	Conditionally essential amino acid that acts as a substrate for nitric oxide synthesis (which is potentially toxic to cancer cells), improves immune function [36,45,67], modulates protein turnover [45], fights remnant cancer cells following surgical removal of malignant tumors [36], and improves wound healing [35,67]. Works in synergy with HMB to mitigate muscle loss, and with both HMB and glutamine to reduce muscle damage from ROS and proinflammatory cytokines [57].	Weight; body composition; LBM; grip strength; energy and protein intake; liver function; renal function; total protein, prealbumin, albumin, globulin, retinol-binding protein, total cholesterol, and triglycerides levels; fatigue; QoL; need for parenteral nutrition during hospital admission; duration of tube feeding; length of hospital stay; fistula incidence after surgery; readmission rates; treatment success; cancer recurrence; metastases or second primary tumors occurrence; survival.
Branched chain amino acids (BCAA)	Improves physical function in elderly patients with advanced lung or pancreatic cancer as part of a multimodal intervention with coQ10 and L-carnitine [56].	Not specified	Weight, BMI, LBM, nutritional status, food intake, physical function/muscle strength, physical activity levels.
Carnitine	Improves cancer cachexia in pancreatic cancer [41], and improves fatigue and ROS levels in advanced cancer [53]. As part of a combined treatment, improves cancer cachexia symptoms [42,44,73,74], cancer-related anemia [73], and physical function in elderly patients with advanced lung or pancreatic cancer [56].	Modulates inflammatory response mechanisms associated with cancer cachexia [41]. Deficiency contributes to cancer cachexia and tumor-associated fatigue [41]. Key in β-oxidation and energy and amino acid metabolism [42,44,53,73].	Weight; BMI; body composition; LBM; skeletal muscle analysis; grip strength; food intake; nutritional status; nutrition impact symptoms; REE; L-carnitine, iron, ferritin, hepcidin, erythropoietin, hemoglobin; CRP, proinflammatory cytokines, ROS, and antioxidant enzyme levels; appetite; fatigue; performance status; physical function; physical activity level; QoL; global health status; prognostic score; survival; anemia; adverse events/toxicity.
Glutamine	As part of a mixture with arginine and HMB, prevents LBM loss and reverses cancer cachexia [35,45] or delays cachexia onset in advanced lung cancer [57].	Regulates muscle protein synthesis or turnover [35,57] and exerts immune stimulatory effects [45]. Works with both HMB and arginine to reduce muscle damage from ROS and pro-inflammatory cytokines [57].	Weight; body composition; LBM; grip strength; calorie and protein intake; liver function; renal function; total protein, albumin, globulin, prealbumin, triglyceries and total cholesterol levels; fatigue; QoL; treatment success.
HMB	As part of a mixture with arginine and glutamine, prevents LBM loss and reverses cancer cachexia [35,45] or delays cachexia onset in advanced lung cancer [57].	Modulates protein turnover [45,57] and works in synergy with arginine to mitigate muscle loss [57]. Improves nitrogen balance, inhibits proteolysis-inducing factor [35], and works with arginine and glutamine to reduce muscle damage from ROS and pro-inflammatory cytokines [57].	Weight; body composition; LBM; grip strength; calorie and protein intake; liver function; renal function; total protein, albumin, globulin and prealbumin levels; triglyceries and total cholesterol levels; fatigue; QoL; treatment success.
**Fatty Acids**			
EPA ± docosahexaenoic acid (DHA)	Modifies membrane composition of neutrophils to reduce inflammation and wasting in advanced cancer [47], improves T-cell subsets and cytokine production when used along with vitamin E [39], and improves SIMS symptoms [37]. Omega-3– or EPA–containing ONS improves nutritional, clinical and inflammatory parameters, and health-related QoL in advanced lung cancer [48]; improves prognosis in advanced gastrointestinal cancer [49] and hypopharyngeal cancer among patients on induction chemotherapy [71]; and improves cachexia during gemcitabine therapy [51]. Improves postoperative recovery in head and neck cancer, as part of an immunonutrition formula [67]. Omega-3–containing ONS as part of a multimodal intervention improves clinical outcomes in cancer [69] and attenuates cachexia in incurable lung or pancreatic cancer [50] or advanced cancer with CACS [44,54]. Marine phospholipids (with >50% phospholipid-bound fatty acids as EPA and DHA) aid in cancer cachexia management [63]. Omega-3 fatty acids (EPA and DHA) in krill oil improve lipid profile disorder and inflammatory processes associated with cachexia [78]. A more purified EPA + DHA supplement is more reliable than fish oil supplements [69]. EPA is a biologically active component of fish oil responsible for anticachectic activity [65].	Immunomodulatory [49,63,65,71], with EPA in particular having anti-inflammatory, anticachectic, antitumoral, anti-genotoxic, and antioxidant properties [48,49,63,65,71]. EPA partially substitutes arachidonic acid, thereby reducing production of arachidonic acid–derived mediators to exert an anti-inflammatory effect [37,63]. Omega-3 augments defense against tumor cells and tumor cell susceptibility by altering cell membrane composition and directly reducing tumor cell proliferation [39]. By inhibiting proinflammatory cytokines production [48,49,63,65,71], omega-3 improves cancer cachexia [44,51,63,65] and reduces proinflammatory mediators to lower infections [67]. As fatty acids bound to phospholipids-bound are more readily incorporated into plasma phospholipids compared to those bound to triacylglycerols, marine phospholipids can be more effective in a lower dose than fish oil in cancer patients [63]. A purified EPA/DHA supplement offers better outcomes due to possible interactions between platinum-based chemotherapy and fatty acids present in fish oils [69].	Weight; height; BMI; fat mass; subcutaneous and visceral adipose tissue; LBM; muscle mass; skeletal muscle; phase angle; intracellular water; extracellular water; total body water; grip strength; REE; nutritional status; nutritional, caloric intake and fat intake; complete blood cell count; white blood cell count; leukocytes, thrombocytes, leptin, retinol-binding protein, albumin, pre-albumin, transferrin, total protein, hemoglobin, glucose, sodium, potassium, ionic calcium, creatinine, urea, total and direct bilirubin, aspartate amino transaminase, alanine amino transaminase, lactate dehydrogenase, gamma-glutamyl transferase; gamma glutamil transpeptidase, alkaline phosphatase, creatinine, CRP, ROS, antioxidant enzyme, cytokine, T-cell subsets, carcino-embryonic antigen, carbohydrate antigen 19.9/125, lyso-phosphatidylcholine. total cholesterol, high-density lipoprotein, low-density lipoprotein, very low–density lipoprotein, and triglycerides levels; plasma phospholipid, red blood cell, mononuclear lymphocytes, and neutrophil fatty acid composition; appetite; nausea; vomiting; diarrhea; energy level; physical and overall well-being; fatigue; physical activity level, physical function; performance status; QoL; health-related QoL; disease progression; prognostic score; survival; toxicity; adverse events; safety; therapy response; chemotherapy dose reductions; need for parenteral nutrition during admission; duration of tube feeding; fistula incidence after surgery; mortality; hospital length of stay; hospitalizations; recurrence; readmissions; adherence to intervention.
Fiber			
Fiber	Not reported	Not reported	Weight, muscle mass, fat-free mass, fat mass; hemoglobin, glucose, CRP, albumin, AAT, GGT, carcinoembryonic antigen, and carbohydrate antigen 19.9/125 levels.
Others			
β-hydroxybutyrate (BHB)	Intake is safe when used while receiving immunotherapy for cancer [81].	Not reported	Weight; sarcopenia; albuminemia; prealbuminemia; CRP level; QoL; response rate; survival; safety.
CoQ10	Improves physical function in elderly patients with advanced lung or pancreatic cancer as part of a multimodal intervention [56].	Not reported	Weight, BMI, skeletal muscle analysis, grip strength, food intake, nutritional status, nutrition impact symptoms, physical function, physical activity levels.
Muscadine grape extract (MGE)	Improves cancer outcomes by reducing symptom burden and is tolerated and safe for use in patients with metastatic solid tumors who have failed standard therapies [52].	Muscadine grape contains a high concentration of anthocyanin, ellagic acid, gallic acid, and flavonols and has antioxidant properties. It inhibits tumor cell growth and induces apoptosis, while also reducing systemic inflammation [52].	CRP, hepatocyte growth factor, IL-6, IL-6 receptor, IL-8, platelet-derived growth factor, TNF-α, vascular endothelial growth factor and phenolic levels; fatigue, QoL, response rate; safety; survival; adherence to intervention.
Dietary nucleotides	Improves postoperative recovery in head and neck cancer as part of an immunonutrition enteral formula [67].	Modulates inflammatory and immune response [67].	Weight, energy and protein intake, albumin levels, retinol binding protein levels, duration of tube feeding, need for parenteral nutrition during admission, fistula incidence after surgery, length of hospital stay, readmission rates, mortality.
Royal jelly	Protects from toxicities induced by tyrosine kinase inhibitors in renal cancer [34].	Possesses anti-inflammatory and antioxidative effects, influences immune system, and protects from adverse events such as inflammation, oxidative stress and immune system dysfunction induced by anticancer agents [34].	Tumor necrosis and transforming growth factor levels, adverse events due to tyrosine kinase inhibitors, sustained period of initial tyrosine kinase inhibitors.

1α-OH vitamin D3: 1α-OHD3; CACS: cancer-related anorexia/cachexia syndrome; ONS: oral nutritional supplements; LBM: lean body mass; REE: resting energy expenditure; ROS: reactive oxygen species; QoL: quality of life; CRP: C-reactive protein; HMB: β-hydroxyl β-methyl butyrate; BMI: body mass index; SIMS: systemic immune-metabolic syndrome; COX: cyclooxygenases; AAT: alanine aminotransferase; GGT: gamma-glutamyl transferase; IL: interleukin; TNF: tumor necrosis factor.

**Table 5:** Outcome measures and corresponding tools used in included studies.

Outcome Measures	Tools Used
Nutritional intake	Two-day or three-day food diary, 24-h diet recall; 10-point verbal scale assessment of nutritional intake [38,50,56,58]
Nutritional status	MNA; PG-SGA [50,56]
Body composition (including fat-free mass, LBM, muscle mass)	Midarm muscle circumference measurement; skin-fold measurement techniques; body plethysmography; air displacement plethysmograph; BIA; BIVA; CT; DEXA [35,37,38,41,42,44,45,48,49,50,53,54,55,56,57,58,63,65,72]
Phase angle	BIVA; BIA [38,48]
Grip strength	Handgrip Dynamometer [38,42,44,50,53,54,56,57]
Muscle strength/physical performance/exercise capacity	Five times sit-to-stand or chair rise test; 5-m gait speed; 10-m gait speed; stair-climbing power; 6-min walk distance [50,56,64,72]
Resting energy expenditure	Indirect calorimetry [42,44,54]
Fatigue	MFSI-SF; Brief Fatigue Inventory questionnaire; Schwarz Fatigue Index; numerical rating scale 0–10; Fatigue Severity Scale; PROMIS-fatigue [35,37,41,42,44,50,52,53,54]
Appetite	VAS; numerical rating scale 0–10 [37,42,44,54,63]
Nausea	Numerical rating scale 0–10 [37]
Performance status	ECOG PS scale; Karnofsky performance status/score [37,39,44,51,54]
Physical activity level	Electronic wearable device (armband/pedometer/accelerometer/logger) [44,50,56]
QoL	EORTC QLQ-C30; EORTC-QLQ-C30 questionnaire with pancreatic cancer–specific module PAN 26; Italian version of EORTC QLQ-C30; QLQ-LC13, EQ-5D; QoL-ACD; FAACT; FACT-L; FACT-G; Functional Assessment Health Survey; Functional Assessment of Cancer Therapy–General as well as –Bone Pain, –Breast and –Endocrine Symptoms subscales; Spitzer Quality of Life Index; Short Form-36 Health Survey; Quality of Life–Oxidative Stress Questionnaire; disease and treatment assessment form [35,38,41,42,44,45,46,48,52,54,57,58,59,63,80,81]
Symptom burden	Brief Pain Inventory; Piper Fatigue Scale; Hospital Anxiety and Depression Scale; and Pittsburg Sleep Quality Index [59]
Pain	Modified McGill–Dartmouth Pain Questionnaire [64]
Prognosis	GPS [44,74]
Regression of cervical adenopathy	Clinical examination; positron emission tomography (PET); CT [70]
Treatment response	CT, magnetic imaging resonance imaging, or X-ray [52,55]
Tumor response	RECIST [46,51,60,61,68,81]
Toxicity or adverse events	National Cancer Institute Common Toxicity Criteria for Adverse Events; National Cancer Institute Common Toxicology Criteria; National Cancer Institute Common Terminology Criteria for Adverse Events version 3.0 or 4.0; National Cancer Institute’s Common Toxicity Criteria version 2.0 [38,42,43,46,48,50,51,53,58,60,61,66,68]

MNA: Mini Nutritional Assessment; aPG-SGA: abridged Patient Generated Subjective Global Assessment; LBM: Lean Body mass; BIA: Bioelectrical Impedance Analysis; BIVA: bioelectrical impedance vector analysis; CT: computed tomography; DEXA: Dual-Energy X-ray Absorptiometry; MFSI-SF: Multidimensional Fatigue Symptom Inventory—Short Form; PROMIS: Patient reported Outcomes Measurement and Information System; VAS: Visual Analog Scale; ECOG PS: Eastern Cooperative Oncology Group Performance Status; EORTC QLQ-C30: European Organization for Research and Treatment of Cancer Quality of Life Questionnaire C30; EQ-5D: EuroQoL 5 Dimension; QoL: Quality of Life; QoL-ACD: Questionnaire for Cancer Patients Treated with Anticancer Drugs; FAACT: Functional Assessment of Anorexia Cachexia Therapy questionnaire; FACT-L: Functional Assessment of Cancer Therapy for Lung Cancer; FACT-G: Functional Assessment of Cancer Therapy-General; GPS: Glasgow Prognostic Score; RECIST: Response Evaluation Criteria in Solid Tumors.

## Data Availability

Supporting data are available upon request. Details of the excluded papers are available from the corresponding author on request.

## Supplementary Materials

The following supporting information can be downloaded at: https://www.mdpi.com/article/10.3390/nu14132642/s1, File S1: PRISMA-ScR Checklist; File S2: Search Strategy; File S3: Data Extraction Form Template.
