# Supplementary material for: Dietary Supplements in People with Metastatic Cancer Who Are Experiencing Malnutrition, Cachexia, Sarcopenia, and Frailty: A Scoping Review
Source: Nutrients. 2022 Jun 26;14(13):2642. doi: 10.3390/nu14132642 (PMC9268679; doi:10.3390/nu14132642)
Supplement: Supplementary file 1 [file nutrients-14-02642-s001.zip › File S2 Search Strategy.pdf]

Dietary supplements in people with metastatic cancers who are experiencing malnutrition, cachexia, sarcopenia, and frailty: a scoping review

Search Strategy

Medline via OVID

Search	Query
#1	(Supplement* OR nutraceutical* OR nutriceutical* OR vitamin* OR antioxidant* OR beta?carotene OR carotene* OR carotenoid* OR retinol OR niacin OR folate OR cobalamin OR pyridoxine OR ascorbic acid OR cholecalciferol OR calcitriol OR tocopherol* OR CoQ10 OR coenzyme Q10 OR mineral* OR zinc OR magnesium OR manganese OR iron OR selenium OR copper OR calcium OR micronutrient* OR amino acid* OR histidine OR lysine OR methionine OR phenylalanine OR threonine OR arginine OR alanine OR glycine OR glutamine OR carnitine OR tryptophan OR leucine OR isoleucine OR valine OR HMB OR hydroxy?methyl?butyrate OR diet* protein* OR whey OR WPI OR fatty acid* OR omega?3 OR n-3 OR EPA OR DHA OR eicosapentaenoic acid* OR docosahexaenoic acid* OR alpha?linolenic acid OR fish oil* OR fiber OR fibre OR prebiotic* OR oligosaccharide* OR probiotic* OR synbiotic*).ti,ab,kw.
#2	Dietary Supplements/ OR exp Vitamins/ OR Vitamin A/ OR Vitamin B 12/ OR Vitamin B 6/ OR Vitamin B Complex/ OR Vitamin D/ OR Vitamin E/ OR Antioxidants/ OR Zinc/ OR Magnesium/ OR Manganese/ OR Iron/ OR Selenium/ OR Copper/ OR Calcium/ OR exp Micronutrients/ OR Amino Acids/ OR Amino Acids, Branched-Chain/ OR Amino Acids, Essential/ OR Histidine/ OR Lysine/ OR Methionine/ OR Phenylalanine/ OR Threonine/ OR Arginine/ OR Alanine/ OR Glycine/ OR Glutamine/ OR Carnitine/ OR Tryptophan/ OR Leucine/ OR Isoleucine/ OR Valine/ OR Dietary Proteins/ OR Whey/ OR Whey Proteins/ OR Fatty Acids/ OR Fatty Acids, Unsaturated/ OR exp Fatty Acids, Omega-3/ OR Eicosapentaenoic Acid/ OR Docosahexaenoic Acids/ OR alpha-Linolenic acid/ OR Fish Oils/ OR exp Dietary Fiber/ OR Prebiotics/ OR Oligosaccharides/ OR Probiotics/ OR Synbiotics/
#3	1 OR 2
#4	(Weight?los* OR malnutrition OR malnourish* OR undernutrition OR undernourish* OR emaciat* OR starv* OR cachexia OR cachectic OR precachexia OR precachectic OR anorexia OR anorexia?cachexia OR CACS OR wasting OR wasted OR muscle loss* OR muscular loss* OR muscle atrophy OR muscular atrophy OR sarcop?enia OR pre?frail* OR frail*).ti,ab,kw.
#5	Weight Loss/ OR exp Malnutrition/ OR Wasting Syndrome/ OR Protein-Energy Malnutrition/ OR Emaciation/ OR Starvation/ OR Cachexia/ OR Anorexia/ OR Muscular Atrophy/ OR Sarcopenia/ OR Frailty/
#6	4 OR 5
#7	(Cancer* OR carcinoma* OR malignan* OR oncology OR tumor* OR tumour* OR neoplasm*).ti,ab,kw.
#8	exp Carcinoma/ OR exp Neoplasms/
#9	7 OR 8
#10	(Recur* OR advanced OR late?stage OR end?stage OR metastatic OR metastas?s OR Stage IV OR Stage 4 OR Stage Four OR incurable OR palliative OR terminal).ti,ab,kw.
#11	Neoplasm Recurrence, Local/ OR Neoplasm Metastasis/ OR Lymphatic Metastasis/

Search	Query
#12	10 OR 11
#13	3 AND 6 AND 9 AND 12
#14	Limit to English Language
#15	Limit to humans

CINAHL via EBSCO

Search	Query
#1	TI (Supplement* OR nutraceutical* OR nutriceutical* OR vitamin* OR antioxidant* OR beta#carotene OR carotene* OR carotenoid* OR retinol OR niacin OR folate OR cobalamin OR pyridoxine OR ascorbic acid OR cholecalciferol OR calcitriol OR tocopherol* OR CoQ10 OR coenzyme Q10 OR mineral* OR zinc OR magnesium OR manganese OR iron OR selenium OR copper OR calcium OR micronutrient* OR amino acid* OR histidine OR lysine OR methionine OR phenylalanine OR threonine OR arginine OR alanine OR glycine OR glutamine OR carnitine OR tryptophan OR leucine OR isoleucine OR valine OR HMB OR hydroxy#methyl#butyrate OR diet* protein* OR whey OR WPI OR fatty acid* OR omega#3 OR n-3 OR EPA OR DHA OR eicosapentaenoic acid* OR docosahexaenoic acid* OR alpha#linolenic acid OR fish oil* OR fiber OR fibre OR prebiotic* OR oligosaccharide* OR probiotic* OR synbiotic*) OR AB (Supplement* OR nutraceutical* OR nutriceutical* OR vitamin* OR antioxidant* OR beta#carotene OR carotene* OR carotenoid* OR retinol OR niacin OR folate OR cobalamin OR pyridoxine OR ascorbic acid OR cholecalciferol OR calcitriol OR tocopherol* OR CoQ10 OR coenzyme Q10 OR mineral* OR zinc OR magnesium OR manganese OR iron OR selenium OR copper OR calcium OR micronutrient* OR amino acid* OR histidine OR lysine OR methionine OR phenylalanine OR threonine OR arginine OR alanine OR glycine OR glutamine OR carnitine OR tryptophan OR leucine OR isoleucine OR valine OR HMB OR hydroxy#methyl#butyrate OR diet* protein* OR whey OR WPI OR fatty acid* OR omega#3 OR n-3 OR EPA OR DHA OR eicosapentaenoic acid* OR docosahexaenoic acid* OR alpha#linolenic acid OR fish oil* OR fiber OR fibre OR prebiotic* OR oligosaccharide* OR probiotic* OR synbiotic*)
#2	(MH “Dietary Supplements+”) OR (MH “Dietary Supplementation”) OR (MH “Vitamins+”) OR (MH “Vitamin A+”) OR (MH “Vitamin B12”) OR (MH “pyridoxine”) OR (MH “Vitamin B Complex+”) OR (MH “Vitamin D+”) OR (MH “Vitamin E”) OR (MH “Antioxidants+”) OR (MH “Zinc”) OR (MH “Magnesium”) OR (MH “Manganese”) OR (MH “Iron”) OR (MH “Selenium”) OR (MH “Copper”) OR (MH “Calcium”) OR (MH “Micronutrients”) OR (MH “Amino Acids+”) OR (MH “Amino Acids, Branched-Chain+”) OR (MH “Amino Acids, Essential+”) OR (MH “Histidine”) OR (MH “Lysine”) OR (MH “Methionine+”) OR (MH “Phenylalanine”) OR (MH “Threonine”) OR (MH “Arginine”) OR (MH “Alanine”) OR (MH “Glycine”) OR (MH “Glutamine”) OR (MH “Carnitine”) OR (MH “Tryptophan”) OR (MH “Leucine”) OR (MH “Isoleucine”) OR (MH “Valine”) OR (MH “Dietary Proteins+”) OR (MH “Whey Proteins”) OR (MH “Fatty Acids+”) OR (MH “Fatty Acids, Unsaturated+”) OR (MH “Fatty Acids, Omega-3+”) OR (MH “Eicosapentaenoic Acid”) OR (MH “Docosahexaenoic Acids”) OR (MH “alpha-Linolenic acid”) OR (MH “Fish Oils+”) OR (MH “Dietary Fiber”) OR (MH “Prebiotics”) OR (MH “Oligosaccharides+”) OR (MH “Probiotics”)
#3	1 OR 2
#4	TI (Weight#los* OR malnutrition OR malnourish* OR undernutrition OR undernourish* OR emaciat* OR starv* OR cachexia OR cachectic OR precachexia OR precachectic OR anorexia OR anorexia#cachexia OR CACS OR wasting OR wasted OR muscle loss* OR muscular loss* OR muscle atrophy OR muscular atrophy OR sarcop#enia OR pre#frail* OR frail*) OR AB (Weight#los* OR malnutrition OR malnourish* OR undernutrition OR

Search	Query
	undernourish* OR emaciat* OR starv* OR cachexia OR cachectic OR precachexia OR precachectic OR anorexia OR anorexia#cachexia OR CACS OR wasting OR wasted OR muscle loss* OR muscular loss* OR muscle atrophy OR muscular atrophy OR sarcop#enia OR pre#frail* OR frail*)
#5	(MH “Weight Loss+”) OR (MH “Malnutrition”) OR (MH “Protein-Energy Malnutrition+”) OR (MH “Wasting Syndrome+”) OR (MH “Whey Proteins”) OR (MH “Starvation”) OR (MH “Cachexia”) OR (MH “Anorexia”) OR (MH “Muscular Atrophy+”) OR (MH “Sarcopenia”) OR (MH “Frailty Syndrome”)
#6	4 OR 5
#7	TI (Cancer* OR carcinoma* OR malignan* OR oncology OR tumor* OR tumour* OR neoplasm*) OR AB (Cancer* OR carcinoma* OR malignan* OR oncology OR tumor* OR tumour* OR neoplasm*)
#8	(MH “Carcinoma+”) OR (MH “Neoplasms+”) OR (MH “Oncology+”)
#9	7 OR 8
#10	TI (Recur* OR advanced OR late#stage OR end#stage OR metastatic OR metastas#s OR Stage IV OR Stage 4 OR Stage Four OR incurable OR palliative OR terminal) OR AB (Recur* OR advanced OR late#stage OR end#stage OR metastatic OR metastas#s OR Stage IV OR Stage 4 OR Stage Four OR incurable OR palliative OR terminal)
#11	(MH “Recurrence+”) OR (MH “Neoplasm Recurrence, Local”) OR (MH “Neoplasm Metastasis+”) OR (MH “Bone Metastases”) OR (MH “Palliative Care”) OR (MH “Terminal Care+”)
#12	10 OR 11
#13	3 AND 6 AND 9 AND 12
#14	Limit to English Language
#15	Limit to humans

Cochrane Library

Search	Query
#1	(Supplement* OR nutraceutical* OR nutriceutical* OR vitamin* OR antioxidant* OR beta*carotene OR carotene* OR carotenoid* OR retinol OR niacin OR folate OR cobalamin OR pyridoxine OR ascorbic acid OR cholecalciferol OR calcitriol OR tocopherol* OR CoQ10 OR coenzyme Q10 OR mineral* OR zinc OR magnesium OR manganese OR iron OR selenium OR copper OR calcium OR micronutrient* OR amino acid* OR histidine OR lysine OR methionine OR phenylalanine OR threonine OR arginine OR alanine OR glycine OR glutamine OR carnitine OR tryptophan OR leucine OR isoleucine OR valine OR HMB OR hydroxy*methyl*butyrate OR diet* protein* OR whey OR WPI OR fatty acid* OR omega*3 OR n-3 OR EPA OR DHA OR eicosapentaenoic acid* OR docosahexaenoic acid* OR alpha*linolenic acid OR fish oil* OR fiber OR fibre OR prebiotic* OR oligosaccharide* OR probiotic* OR synbiotic*):ti,ab,kw.
#2	[mh “Dietary Supplements”] OR [mh “Vitamins”] OR [mh “Vitamin A”] OR [mh “Vitamin B 12”] OR [mh “Vitamin B 6”] OR [mh “Vitamin B Complex”] OR [mh “Vitamin D”] OR [mh “Vitamin E”] OR [mh “Antioxidants”] OR [mh “Zinc”] OR [mh “Magnesium”] OR [mh “Manganese”] OR [mh “Iron”] OR [mh “Selenium”] OR [mh “Copper”] OR [mh “Calcium”] OR [mh “Micronutrients”] OR [mh “Amino Acids”] OR [mh “Amino Acids, Branched-Chain”] OR [mh “Amino Acids, Essential”] OR [mh “Histidine”] OR [mh “Lysine”] OR [mh “Methionine”] OR [mh “Phenylalanine”] OR [mh “Threonine”] OR [mh “Arginine”] OR [mh “Alanine”] OR [mh “Glycine”] OR [mh “Glutamine”] OR [mh “Carnitine”] OR [mh “Tryptophan”] OR [mh “Leucine”] OR [mh “Isoleucine”] OR [mh “Valine”] OR [mh “Dietary Proteins”] OR [mh “Whey”] OR [mh “Whey Proteins”] OR

Search	Query
	[mh “Fatty Acids”] OR [mh “Fatty Acids, Unsaturated”] OR [mh “Fatty Acids, Omega-3”] OR [mh “Eicosapentaenoic Acid”] OR [mh “Docosahexaenoic Acids”] OR [mh “alpha-Linolenic acid”] OR [mh “Fish Oils”] OR [mh “Dietary Fiber”] OR [mh “Prebiotics”] OR [mh “Oligosaccharides”] OR [mh “Probiotics”] OR [mh “Synbiotics”]
#3	#1 OR #2
#4	(Weight*los* OR malnutrition OR malnourish* OR undernutrition OR undernourish* OR emaciat* OR starv* OR cachexia OR cachectic OR precachexia OR precachectic OR anorexia OR anorexia*cachexia OR CACS OR wasting OR wasted OR muscle loss* OR muscular loss* OR muscle atrophy OR muscular atrophy OR sarcop*enia OR pre*frail* OR frail*):ti,ab,kw.
#5	[mh “Weight Loss”] OR [mh “Malnutrition”] OR [mh “Wasting Syndrome”] OR [mh “Protein-Energy Malnutrition”] OR [mh “Emaciation”] OR [mh “Starvation”] OR [mh “Cachexia”] OR [mh “Anorexia”] OR [mh “Muscular Atrophy”] OR [mh “Sarcopenia”] OR [mh “Frailty”]
#6	#4 OR #5
#7	(Cancer* OR carcinoma* OR malignan* OR oncology OR tumor* OR tumour* OR neoplasm*):ti,ab,kw.
#8	[mh “Carcinoma”] OR [mh “Neoplasms”]
#9	#7 OR #8
#10	(Recur* OR advanced OR late*stage OR end*stage OR metastatic OR metastas*s OR Stage IV OR Stage 4 OR Stage Four OR incurable OR palliative OR terminal):ti,ab,kw.
#11	[mh “Neoplasm Recurrence, Local”] OR [mh “Neoplasm Metastasis”] OR [mh “Lymphatic Metastasis”]
#12	#10 OR #11
#13	#3 AND #6 AND #9 AND #12
#14	Limit to English Language
#15	Limit to humans

JBI EBP Database via OVID (Includes JBI Evidence Synthesis Journal)

Search	Query
#1	(Supplement* OR nutraceutical* OR nutriceutical* OR vitamin* OR antioxidant* OR beta?carotene OR carotene* OR carotenoid* OR retinol OR niacin OR folate OR cobalamin OR pyridoxine OR ascorbic acid OR cholecalciferol OR calcitriol OR tocopherol* OR CoQ10 OR coenzyme Q10 OR mineral* OR zinc OR magnesium OR manganese OR iron OR selenium OR copper OR calcium OR micronutrient* OR amino acid* OR histidine OR lysine OR methionine OR phenylalanine OR threonine OR arginine OR alanine OR glycine OR glutamine OR carnitine OR tryptophan OR leucine OR isoleucine OR valine OR HMB OR hydroxy?methyl?butyrate OR diet* protein* OR whey OR WPI OR fatty acid* OR omega?3 OR n-3 OR EPA OR DHA OR eicosapentaenoic acid* OR docosahexaenoic acid* OR alpha?linolenic acid OR fish oil* OR fiber OR fibre OR prebiotic* OR oligosaccharide* OR probiotic* OR synbiotic*).ti,ab,kw.
#2	(Weight?los* OR malnutrition OR malnourish* OR undernutrition OR undernourish* OR emaciat* OR starv* OR cachexia OR cachectic OR precachexia OR precachectic OR anorexia OR anorexia?cachexia OR CACS OR wasting OR wasted OR muscle loss* OR muscular loss* OR muscle atrophy OR muscular atrophy OR sarcop?enia OR pre?frail* OR frail*).ti,ab,kw.
#3	(Cancer* OR carcinoma* OR malignan* OR oncology OR tumor* OR tumour* OR neoplasm*).ti,ab,kw.

Search	Query
#4	(Recur* OR advanced OR late?stage OR end?stage OR metastatic OR metastas?s OR Stage IV OR Stage 4 OR Stage Four OR incurable OR palliative OR terminal).ti,ab,kw.

Embase via OVID

Search	Query
#1	(Supplement* OR nutraceutical* OR nutriceutical* OR vitamin* OR antioxidant* OR beta?carotene OR carotene* OR carotenoid* OR retinol OR niacin OR folate OR cobalamin OR pyridoxine OR ascorbic acid OR cholecalciferol OR calcitriol OR tocopherol* OR CoQ10 OR coenzyme Q10 OR mineral* OR zinc OR magnesium OR manganese OR iron OR selenium OR copper OR calcium OR micronutrient* OR amino acid* OR histidine OR lysine OR methionine OR phenylalanine OR threonine OR arginine OR alanine OR glycine OR glutamine OR carnitine OR tryptophan OR leucine OR isoleucine OR valine OR HMB OR hydroxy?methyl?butyrate OR diet* protein* OR whey OR WPI OR fatty acid* OR omega?3 OR n-3 OR EPA OR DHA OR eicosapentaenoic acid* OR docosahexaenoic acid* OR alpha?linolenic acid OR fish oil* OR fiber OR fibre OR prebiotic* OR oligosaccharide* OR probiotic* OR synbiotic*).ti,ab,kw.
#2	Dietary Supplement/ OR Vitamin/ OR retinol/ OR cyanocobalamin/ OR pyridoxine/ OR vitamin B complex/ OR vitamin D/ OR alpha tocopherol/ OR antioxidant/ OR zinc/ OR Magnesium/ OR Manganese/ OR Iron/ OR Selenium/ OR Copper/ OR Calcium/ OR trace element/ OR amino acid/ OR branched chain amino acid/ OR essential amino acid/ OR histidine/ OR Lysine/ OR Methionine/ OR Phenylalanine/ OR Threonine/ OR Arginine/ OR Alanine/ OR Glycine/ OR Glutamine/ OR Carnitine/ OR Tryptophan/ OR Leucine/ OR Isoleucine/ OR Valine/ OR protein intake/ OR whey/ OR whey protein/ OR fatty acid/ OR unsaturated fatty acid/ OR omega 3 fatty acid/ OR icosapentaenoic acid/ OR Docosahexaenoic Acids/ OR linolenic acid/ OR fish oil/ OR dietary fiber/ OR prebiotic agent/ OR oligosaccharide/ OR probiotic agent/ OR synbiotic agent/
#3	1 OR 2
#4	(Weight?los* OR malnutrition OR malnourish* OR undernutrition OR undernourish* OR emaciat* OR starv* OR cachexia OR cachectic OR precachexia OR precachectic OR anorexia OR anorexia?cachexia OR CACS OR wasting OR wasted OR muscle loss* OR muscular loss* OR muscle atrophy OR muscular atrophy OR sarcop?enia OR pre?frail* OR frail*).ti,ab,kw.
#5	Body weight Loss/ OR Malnutrition/ OR protein calorie malnutrition/ OR wasting syndrome/ OR emaciation/ OR starvation/ OR cachexia/ OR anorexia/ OR muscle atrophy/ OR sarcopenia/ OR frailty/
#6	4 OR 5
#7	(Cancer* OR carcinoma* OR malignan* OR oncology OR tumor* OR tumour* OR neoplasm*).ti,ab,kw.
#8	Malignant neoplasm/ OR carcinoma/ OR oncology/ OR neoplasm/
#9	7 OR 8
#10	(Recur* OR advanced OR late?stage OR end?stage OR metastatic OR metastas?s OR Stage IV OR Stage 4 OR Stage Four OR incurable OR palliative OR terminal).ti,ab,kw.
#11	Advanced cancer/ OR metastasis/ OR terminal disease/
#12	10 OR 11
#13	3 AND 6 AND 9 AND 12
#14	Limit to English Language
#15	Limit to humans

WEB OF SCIENCE

Search	Query
#1	**TOPIC** (equivalent to searching of title, abstract, author keywords, and Keywords Plus): Supplement* OR nutraceutical* OR nutriceutical* OR vitamin* OR antioxidant* OR beta*carotene OR carotene* OR carotenoid* OR retinol OR niacin OR folate OR cobalamin OR pyridoxine OR ascorbic acid OR cholecalciferol OR calcitriol OR tocopherol* OR CoQ10 OR "coenzyme Q10" OR mineral* OR zinc OR magnesium OR manganese OR iron OR selenium OR copper OR calcium OR micronutrient* OR amino acid* OR histidine OR lysine OR methionine OR phenylalanine OR threonine OR arginine OR alanine OR glycine OR glutamine OR carnitine OR tryptophan OR leucine OR isoleucine OR valine OR HMB OR hydroxy*methyl*butyrate OR diet* protein* OR whey OR WPI OR fatty acid* OR omega*3 OR n-3 OR EPA OR DHA OR eicosapentaenoic acid* OR docosahexaenoic acid* OR alpha*linolenic acid OR "fish oil*" OR fiber OR fibre OR prebiotic* OR oligosaccharide* OR probiotic* OR synbiotic*
#2	**TOPIC:** Weight*los* OR malnutrition OR malnourish* OR undernutrition OR undernourish* OR emaciat* OR starv* OR cachexia OR cachectic OR precachexia OR precachectic OR anorexia OR anorexia*cachexia OR CACS OR wasting OR wasted OR muscle loss* OR muscular loss* OR muscle atrophy OR muscular atrophy OR sarcop*enia OR pre*frail* OR frail*
#3	**TOPIC:** Cancer* OR carcinoma* OR malignan* OR oncology OR tumor* OR tumour* OR neoplasm*
#4	**TOPIC:** Recur* OR advanced OR late*stage OR end*stage OR metastatic OR metastas*s OR "Stage IV" OR "Stage 4" OR "Stage Four" OR incurable OR palliative OR terminal
#5	1 AND 2 AND 3 AND 4
#6	Limit to English

SCOPUS

Search	Query
#1	TITLE-ABS-KEY(Supplement* OR nutraceutical* OR nutriceutical* OR vitamin* OR antioxidant* OR beta*carotene OR carotene* OR carotenoid* OR retinol OR niacin OR folate OR cobalamin OR pyridoxine OR ascorbic acid OR cholecalciferol OR calcitriol OR tocopherol* OR CoQ10 OR "coenzyme Q10" OR mineral* OR zinc OR magnesium OR manganese OR iron OR selenium OR copper OR calcium OR micronutrient* OR amino acid* OR histidine OR lysine OR methionine OR phenylalanine OR threonine OR arginine OR alanine OR glycine OR glutamine OR carnitine OR tryptophan OR leucine OR isoleucine OR valine OR HMB OR hydroxy*methyl*butyrate OR diet* protein* OR whey OR WPI OR fatty acid* OR omega*3 OR n-3 OR EPA OR DHA OR eicosapentaenoic acid* OR docosahexaenoic acid* OR alpha*linolenic acid OR "fish oil*" OR fiber OR fibre OR prebiotic* OR oligosaccharide* OR probiotic* OR synbiotic*)
#2	TITLE-ABS-KEY(Weight*los* OR malnutrition OR malnourish* OR undernutrition OR undernourish* OR emaciat* OR starv* OR cachexia OR cachectic OR precachexia OR precachectic OR anorexia OR anorexia*cachexia OR CACS OR wasting OR wasted OR muscle loss* OR muscular loss* OR muscle atrophy OR muscular atrophy OR sarcop*enia OR pre*frail* OR frail*)
#3	TITLE-ABS-KEY(Cancer* OR carcinoma* OR malignan* OR oncology OR tumor* OR tumour* OR neoplasm*)

Search	Query
#4	TITLE-ABS-KEY(Recur* OR advanced OR late*stage OR end*stage OR metastatic OR metastas*s OR "Stage IV" OR "Stage 4" OR "Stage Four" OR incurable OR palliative OR terminal)
#5	#1 AND #2 AND #3 AND #4
#6	Limit to English

ISRCTN REGISTRY

Search	Query
#1	(Supplement OR supplements OR nutraceutical OR nutraceuticals OR nutriceutical OR nutriceuticals OR vitamin OR vitamins OR antioxidant OR antioxidants OR mineral OR minerals OR micronutrient OR micronutrients OR "amino acid" OR "amino acids" OR arginine OR glutamine OR carnitine OR leucine OR isoleucine OR HMB OR "dietary protein" OR whey OR "fatty acids" OR "omega 3" OR "omega-3" OR EPA OR DHA OR "fish oil" OR "fish oils" OR fiber OR fibre OR prebiotic OR probiotic) AND ("Weight loss" OR malnutrition OR malnourished OR undernutrition OR undernourished OR emaciation OR emaciated OR starvation OR cachexia OR cachectic OR precachexia OR precachectic OR anorexia OR "anorexia cachexia" OR "anorexia-cachexia" OR wasting OR wasted OR "muscle loss" OR "muscle losses" OR sarcopenia OR sarcopaenia OR frail OR frailty) AND (Cancer OR cancers OR carcinoma OR carcinomas OR malignant OR malignancy OR oncology OR tumor OR tumors OR tumour OR tumours OR neoplasm OR neoplasms) AND (advanced OR "late stage" OR "late-stage" OR "end stage" OR "end-stage" OR metastatic OR metastasis OR metastases OR "Stage IV" OR "Stage 4" OR "Stage Four" OR incurable OR palliative OR terminal)

CLINICALTRIALS.GOV

Search	Query
#1	(Cancer OR cancers OR carcinoma OR carcinomas OR malignant OR malignancy OR oncology OR tumor OR tumors OR tumour OR tumours OR neoplasm OR neoplasms)
#2	(Supplements OR vitamin OR minerals OR micronutrients OR "amino acids" OR "fish oils" OR fiber OR fibre) AND ("weight loss" OR malnutrition OR cachexia OR anorexia OR sarcopenia OR frailty) AND (advanced OR metastatic OR metastasis OR metastases)
#3	#1 AND #2

175 Results

World Health Organization International Clinical Trials Registry Platform (WHO ICTRP)

Search	Query
#1	Supplement* AND weight* AND advanced AND cancer* OR Supplement* AND maln* AND advanced AND cancer* OR Supplement* AND cache* AND advanced AND cancer* OR Supplement* AND anorexi* AND advanced AND cancer* OR Supplement* AND sarcopenia* AND advanced AND cancer* OR Supplement* AND frail* AND advanced AND cancer* OR vitamin* AND weight* AND advanced AND cancer* OR mineral* AND weight* AND advanced AND cancer* OR mineral* AND weight* AND advanced AND cancer* OR micronutrient* AND weight* AND advanced AND cancer* OR amino acid* AND weight* AND advanced AND cancer* OR omega* AND weight* AND advanced AND cancer* OR fish oil* AND weight* AND advanced AND cancer* OR fiber* AND weight* AND advanced AND cancer* OR fibre* AND weight* AND advanced AND cancer*
